# Validation of fully automated intensity-modulated proton therapy with and without transmission beams for nasopharyngeal cancer

**DOI:** 10.1016/j.phro.2025.100831

**Published:** 2025-08-30

**Authors:** Merle Huiskes, Wens Kong, Sebastiaan Breedveld, Koen Crama, Martin de Jong, Steven Habraken, Ben Heijmen, Coen Rasch, Eleftheria Astreinidou

**Affiliations:** a Department of Radiation Oncology, Leiden University Medical Center, Leiden, the Netherlands; b Department of Radiotherapy, Erasmus MC Cancer Institute, Rotterdam, the Netherlands; c HollandPTC, Delft, the Netherlands

**Keywords:** Head and neck cancer, Nasopharyngeal cancer, Intensity Modulated Proton Therapy (IMPT), Transmission beams, Automated planning, Multi-criterial optimization (MCO)

## Abstract

**Background and purpose:**

Intensity-modulated proton therapy (IMPT) offers dose/volume advantages for nasopharyngeal cancer (NPC), however is complex, time-consuming, and the Bragg-peak’s broad lateral penumbra can compromise target coverage near critical structures. Transmission proton beams (TBs) may mitigate this, while fully automated planning can ensure high-quality plans with reduced planning times. This study aimed to evaluate the added value of TBs in robust, fully automated IMPT (autoIMPT) planning for NPC, and benchmarking these against manually generated clinical IMPT plans.

**Materials and Methods:**

Twenty-four NPC patients previously treated with IMPT with 70.00 Gy(RBE) to the primary tumor and 54.25 Gy(RBE) to the elective volumes were included. AutoIMPT- and autoIMPT+TB plans were generated by Erasmus-iCycle, using the same 6-beam configuration and clinical constraints. Dose/volume-based comparison to clinical plans was performed for target coverage, OAR doses, and normal tissue complication probabilities (NTCP).

**Results:**

Voxel-wise (vw) minimum target D98% of automated plans was similar or improved compared to clinical plans, especially in cases with compromised target coverage. In serial OARs, significant dose reductions were observed, especially in brainstem and optical system. Dose reductions to serial OARs were more pronounced in the autoIMPT+TB plans, up to 22.5 Gy for vw-maximum optical nerve D0.03cm^3^. Similarly, dose reductions in most parallel OARs were observed (−13.2 Gy in pituitary Dmean). NTCP for dysphagia remained equal; xerostomia NTCP was significantly lower in the automated plans.

**Conclusions:**

Fully automated IMPT and IMPT+TB plans achieved similar or improved robust target coverage and OAR sparing compared to clinical plans. Incorporation of TBs to IMPT showed similar target coverage and enhanced dose reductions in serial OARs.

## Highlights

• Automated proton therapy generated high-quality plans for nasopharyngeal cancer.
• Target coverage and organ-at-risk doses were similar or improved.
• Transmission beams provided additional dose sparing to critical structures.

## Graphical abstract

**Figure:**
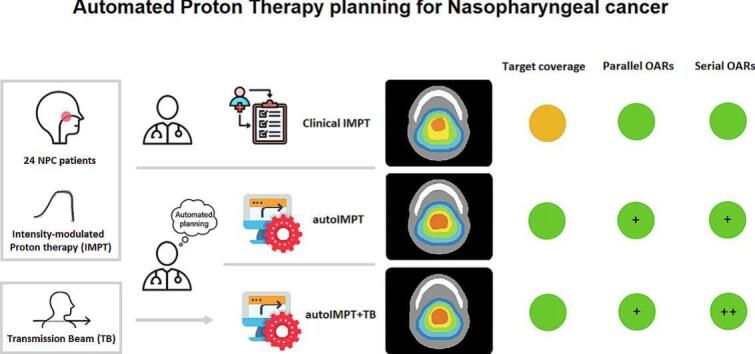

## 1 Introduction

Radiation therapy is a standard curative treatment option for nasopharyngeal cancer (NPC) patients, with concomitant chemotherapy for advanced stages. Intensity modulated proton therapy (IMPT) is increasingly adopted as a treatment modality for NPC, due to its potential to further reduce acute and late toxicity [1,2].

In IMPT, beams have a characteristic sharp distal dose fall off, the Bragg peak, which is generally placed inside the tumor target, resulting in minimal dose beyond the target. However, proton beams have a relatively large lateral penumbra at larger depths as compared to its distal edge penumbra [3]. Also, at lower energies used for superficial dose deposition, and with the addition of range shifters, the lateral penumbra of a proton beam is wider [4]. While for NPC patients the steep distal dose fall off in IMPT allows for more optimal target coverage with reduced dose to surrounding organs at risk (OARs) as compared to photons [5,6], the lateral penumbra width may negatively impact the dose distribution in the lateral direction. The latter is especially relevant at the boundaries of the tumor with serial OARs, where in some cases target coverage concessions are necessary to prevent exceedance of dose constraints to serial OARs, i.e. the brainstem and optic system.

A solution for this may be the incorporation of shoot-through proton beams, or so-called transmission beams (TBs) which are single, high-energy beams which shoot-through the patient. With this technique, the Bragg peak locations end up behind the patient, and the tissue will be irradiated with the part of the beam proximal to the Bragg peak. Advantages of TB proton therapy are sharp lateral penumbras, increased robustness in the beam direction due to less sensitivity to density changes, and no increase in linear energy transfer (LET) in the trajectory. However, despite the possible advantages of TB plans, research on this subject is limited. In proton FLASH, TB have been used [[7], [8], [9]]. Only one head-and-neck study by van Marlen et al.[10] shown that 10-beam TB plans deliver similar quality plans as 3-beam IMPT plans. Nevertheless, a drawback of using TBs alone is the increased exposure of healthy tissues beyond the target, and does not fully leverage the degrees of freedom for dose shaping. Therefore the combination of IMPT with TB is particularly relevant, as transmission beams may enhance target coverage and OAR sparing due to their sharper lateral penumbras.

There is increasing interest in automation in radiotherapy planning, aiming to improve consistency, efficiency, and plan quality while reducing the time and expertise required for manual planning [11,12]. In proton therapy, several automated planning techniques have been developed, including knowledge- based planning [13], hierarchical optimization [14], scripting [15], deep learning [16], and multi-criterial optimization (MCO) [[17], [18], [19]]. The latter is implemented in our in-house planning system as ‘SISS-MCO’ for fully automated robust IMPT planning, which, by design, yields Pareto-optimal plans [20,21].

Previously, a planning strategy for hypo- and oropharynx IMPT was developed using this approach, resulting in superior plan quality as compared to clinical IMPT plans [19]. However, in NPC, conflicting dose constraints between serial OARs and targets can occur, requiring the development of a dedicated planning strategy.

The aim of this study was twofold: (1) to evaluate the added value of incorporating transmission beams (TB) into robust IMPT planning for NPC patients, and (2) to validate fully automated robust IMPT planning for NPC patients. To this purpose, fully automated robust IMPT (autoIMPT) plans and fully automated transmission beams plans in combination with IMPT (autoIMPT+TB) were generated for NPC patients and benchmarked to manually generated clinical IMPT plans. To our knowledge, this is the first dose/volume-based comparison of automated IMPT and IMPT+TB plans for NPC patients with clinical IMPT plans reported in the literature.

## 2 Methods and materials

### 2.1 Patients

All 24 patients with NPC, clinically treated in HollandPTC with primary IMPT at a Varian ProBeam unit (Varian Medical Systems, Palo Alto, CA) between March 2019 and March 2024, were retrospectively included. All patients gave informed consent for the retrospective use of their clinical data. Patients were planned with curative intent and irradiated with 70.00 Gy (relative biological effectiveness (RBE) = 1.1) to the primary Clinical Target Volume (CTV7000), and 54.25 Gy (RBE) to the bilateral elective CTVs (CTV5425) in 35 fractions. Patient and tumor characteristics are shown in Table 1.

**Table 1:** Patient and tumor characteristics.

		Test patients (n = 21)	Tuning patients (n = 3)
Age (years)	Median (range)	48 (18–78)	62 (48–73)
Sex (n)	Female	7	−
	Male	14	3
CTV7000 volume (cm^3^)	Median (range)	156.9 (27.3–408.7)	110.1 (52.5–114.3)
CTV5425 volume (cm^3^)	Median (range)	385.1 (256.6–565.3)	360.6 (333.7–524.7)
T-stage (n)	T1	9	1
	T2	6	1
	T3	5	1
	T4	1	−
N-stage (n)	N0	1	1
	N1	5	2
	N2	11	−
	N3	4	−

### 2.2 Clinical IMPT planning

The clinical IMPT plans were manually generated by an experienced treatment planner in the clinical treatment planning system (RayStation v10B, RaySearch Laboratories AB, Stockholm, Sweden) in accordance with the clinical treatment planning goals (Table S1, Supplementary Material). CTVs and OARs were delineated on a CT with slice thickness of 2x2x2mm^3^. The plans were created with a 6-beam configuration; B1:50°, B2:100°, B3:160°, B4:200°, B5:260°, B6:310°, with a 3 cm range shifter for beams B2-B5. For B2 and B5, a couch rotation of respectively 340° and 20° was applied. Avoidance volumes were created to prevent spots passing through the shoulders, sinuses, and dental fillings. Additionally, caudal from the parotids, the CTV5425 was split into a left- and right part and only allowed to be irradiated with B1-B3 or B4-B6 respectively, to prevent unnecessary irradiation by traversing healthy tissues. The maximum beam contribution was limited to 47.0 Gy per beam. Plans were robustly optimized for 21 scenarios with 3 mm isotropic setup uncertainty and ± 3% range uncertainty[22]. In some cases, to prevent exceedance of constraints to critical OARs, target coverage concessions were made in consultation with the treating physician. Doses were computed with Monte Carlo dose engine on a 3x3x3mm^3^ dose grid with 1.0% statistical uncertainty.

### 2.3 Automated IMPT and IMPT+TB planning

The autoIMPT and autoIMPT+TB plans were generated using ‘SISS-MCO’ (sparsity-induced spot selection with multi-criterial optimization), implemented in the Erasmus-iCycle system for robust IMPT planning[18,19]. The same CT with delineations, 6-beam configuration, and constraints as used clinically was applied. The SISS-MCO framework for IMPT was extended to enable the incorporation of TBs to IMPT planning [23]. Briefly, all selected IMPT spots are duplicated, with their energies replaced by 244 MeV with no range shifters. During the MCO, the optimizer subsequently selects the spots to include in the final plan, see Kong et al. [23].

A nasopharynx specific planning strategy (‘wish-list’) was developed for the purpose of this study (Table S2 in the Supplementary Material). Of the 24 included NPC patients, three were randomly selected for the iterative tuning of the wish-list, in accordance with the clinical protocol. During this process, plans were evaluated, and the wish-list was updated whenever further improvements were considered feasible, see the appendix of Heijmen et al. [24] for further details.

The nasopharynx specific wish-list was applied to the remaining 21 ‘test’ patients for the automated generation of IMPT- and IMPT+TB plans. Doses were computed with the Astroid dose algorithm [25], which was configured for the Varian ProBeam clinical beam characteristics [17].

### 2.4 Plan evaluations and comparisons

The automated plans were evaluated based on the clinical treatment planning goals (Table 2) and compared to the manually generated clinical IMPT plans. Target coverage D98% to the CTVs was reported in the nominal dose and in the voxel-wise minimum (vw-min) dose. Conformity indexes of the CTVs for the 95% isodose were calculated [26]. For the serial OARs, D0.03cm or D2% were evaluated for the nominal dose and voxel-wise maximum (vw-max) dose. Vw-min and vw-max dose distributions were constructed from 28 scenarios with 3 mm setup error and ±3% range uncertainty, the same as in clinical plan evaluations [27]. The mean dose (Dmean) to the parallel OARs was computed from the nominal plans. The V5Gy to the body excluding all CTVs (Body-CTVs) was reported to access the overall dose to the surrounding tissues. The maximum dose per beam was also evaluated. Normal tissue complication probability (NTCP) for xerostomia- and dysphagia (grade ≥ 2 and grade ≥ 3) were calculated, according to the National Indication Protocol Proton Therapy version 2.2, see appendix Table A3 [28]. Wilcoxon signed rank tests were performed to assess statistical significance of observed differences between automated and clinical plans (p < 0.05).

**Table 2:** Dose/volume metrics (median and range) for the CTVs and OARs and the predicted NTCPs for the clinical, automated IMPT- and IMPT + TB plans, with outcome of the Wilcoxon-signed rank test.

	**Metric**+	**Clinical plan** median (min–max)	**autoIMPT plan** median (min–max)	**p-value** **(autoIMPT-Clinical)**	**autoIMPT+TB plan** median (min–max)	**p-value** **(autoIMPT+TB-Clinical)**	**p-value** **(autoIMPT+TB-autoIMPT)**
**CTV7000**	D_98%≥95%_ (66.5 [Gy])	69.0 (65.3–69.4)	68.3 (66.9–68.8)	.04*	68.2 (66.8–68.7)	.02*	.01*
	vw-min D_98%≥94%_ (65.8[Gy])	65.9 (60.8–67.1)	65.9 (62.2–66.3)	.64	65.9 (62.3–66.3)	.61	.93
	D_2%≤107%_ (74.9 [Gy])	73.9 (72.9–74.8)	73.6 (72.7–74.1)	.03*	73.6 (73.1–74.0)	.04*	.99
	Conformity index	0.60 (0.46–0.67)	0.65 (0.60–0.73)	<.001*	0.65 (0.60–0.73)	.00*	.22
**CTV5425**	D_98%≥95%_ (51.5 [Gy])	53.8 (53.3–55.1)	54.1 (53.1–56.1)	.00*	54.2 (53.0–56.2)	.02*	.13
	vw-min D_98%≥94%_ (51.0[Gy])	51.1 (50.4–52.0)	51.4 (50.3–53.0)	.31	51.3 (50.1–52.8)	.39	.02*
	Conformity index	0.49 (0.39–0.62)	0.56 (0.49–0.64)	<.001*	0.56 (0.49–0.65)	<.001*	.02*
**Brainstem**	Core ${\text{D}}_{\text{0.03}{\text{cm}}^{\text{3}}}$[Gy]	33.3 (11.1–53.6)	23.5 (3.9–57.0)	.00*	19.4 (3.5–57.6)	<.001*	.13
	vw-max Core ${\text{D}}_{\text{0.03}{\text{cm}}^{\text{3}}}$[Gy]	46.1 (20.0–59.1)	37.6 (12.9–59.8)	.00*	36.1 (10.1–59.7)	<.001*	.00*
	Surface ${\text{D}}_{\text{0.03}{\text{cm}}^{\text{3}}}$[Gy]	43.2 (17.0–58.2)	31.2 (7.9–62.1)	<.001*	28.1 (7.8–62.0)	<.001*	.04*
	vw-max Surface ${\text{D}}_{\text{0.03}{\text{cm}}^{\text{3}}}$[Gy]	54.4 (26.8–64.0)	47.8 (20.3–64.1)	<.001*	45.5 (19.0–64.1)	<.001*	<.001*
**Spinal cord**	Core ${\text{D}}_{\text{0.03}{\text{cm}}^{\text{3}}}$[Gy]	15.7 (6.6–32.2)	10.2 (4.5–33.9)	.05*	10.1 (3.9–36.1)	.02*	.5
	vw-max Core ${\text{D}}_{\text{0.03}{\text{cm}}^{\text{3}}}$[Gy]	24.0 (96.8–46.9)	14.1 (6.9–41.4)	.00*	14.0 (6.5–45.1)	.00*	.99
	Surface ${\text{D}}_{\text{0.03}{\text{cm}}^{\text{3}}}$ [Gy]	21.5 (7.8–46.6)	12.9 (4.5–4.2)	.00*	12.9 (6.1–44.4)	.00*	.19
	vw-max Surface ${\text{D}}_{\text{0.03}{\text{cm}}^{\text{3}}}$[Gy]	27.5 (12.7–59.6)	17.2 (9.6–51.6)	<.001*	15.7 (8.3–54.2)	<.001*	.99
**Optic chiasm**	${\text{D}}_{\text{0.03}{\text{cm}}^{\text{3}}}$[Gy]	7.6 (1.5–55.3)	0.5 (0–57.4)	<.001*	0.2 (0–57.7)	<.001*	.00*
	vw-max ${\text{D}}_{\text{0.03}{\text{cm}}^{\text{3}}}$[Gy]	13.4 (2.8–59.1)	2.0 (0–59.7)	<.001*	1.0 (0–59.6)	<.001*	.00*
**Optic nerve left**	${\text{D}}_{\text{0.03}{\text{cm}}^{\text{3}}}$[Gy]	25.4 (1.0–70.1)	6.7 (1–57.8)	<.001*	3.9 (0–58.2)	<.001*	.00*
	vw-max ${\text{D}}_{\text{0.03}{\text{cm}}^{\text{3}}}$[Gy]	35.9 (1.9–71.1)	18.4 (5–60.0)	<.001*	13.5 (0–60.3)	<.001*	.00*
**Optic nerve right**	${\text{D}}_{\text{0.03}{\text{cm}}^{\text{3}}}$[Gy]	25.6 (0.7–57.2)	7.2 (0–53.7)	.00*	4.4 (0–53.9)	<.001*	.01*
	vw-max ${\text{D}}_{\text{0.03}{\text{cm}}^{\text{3}}}$[Gy]	35.0 (1.1–59.2)	17.4 (1–57.6)	.00*	12.5 (0–56.5)	<.001*	.00*
**Eye left**	D_mean_ [Gy]	1.0 (0.2–11.2)	0.1 (0–11.2)	.00*	0 (0–9.5)	.00*	.09
**Eye right**	D_mean_ [Gy]	0.6 (0.2–8.8)	0 (0–19.6)	.01*	0 (0–17.8)	.02*	.29
**Lens left**	${\text{D}}_{\text{0.03}{\text{cm}}^{\text{3}}}$[Gy]	0.9 (0.2–9.2)	0 (0–4.7)	<.001*	0 (0–7.4)	<.001*	.41
	vw-max ${\text{D}}_{\text{0.03}{\text{cm}}^{\text{3}}}$[Gy]	1.9 (0.3–11.8)	0 (0–9.6)	<.001*	0 (0–8.9)	<.001*	.08*
**Lens right**	${\text{D}}_{\text{0.03}{\text{cm}}^{\text{3}}}$[Gy]	0.4 (0.2–9.0)	0 (0–19.3)	.01*	0 (0–17.8)	.04*	.53
	vw-max ${\text{D}}_{\text{0.03}{\text{cm}}^{\text{3}}}$[Gy]	0.5 (0.2–9.7)	0 (0–18.4)	.01*	0 (0–18.6)	.01*	.46*
**Brain-CTV7000**	${\text{D}}_{\text{0.03}{\text{cm}}^{\text{3}}}$[Gy]	67.0 (36.1–76.5)	63.4 (30.2–69.2)	<.001*	63.1 (30.2–69.2)	<.001*	.64
	vw-max ${\text{D}}_{\text{3}{\text{cm}}^{\text{3}}}$ [Gy]	58.5 (27.1–71.3)	56.0 (24.9–68.8)	<.001*	56.3 (25.3–68.9)	.00*	.18
	V_10Gy_ [cm^3^]	120 (43–382)	110 (26–357)	<.001*	105 (22–371)	.00*	.05*
**Pituitary**	D_mean_ [Gy]	19.8 (2.3–62.1)	11.0 (0–65.0)	.00*	6.6 (0–65.0)	.00*	.00*
**Cochlea contra**	D_mean_ [Gy]	9.4 (1.6–32.7)	7.1 (0.7–41.4)	.07	4.8 (0.9–36.0)	.02*	.00*
**Cochlea ipsi**	D_mean_ [Gy]	23.0 (6.0–69.1)	14.9 (1.9–67.0)	.01*	11.4 (2.1–64.9)	.00*	.02*
**Parotid contra**	D_mean_ [Gy]	17.4 (6.5–37.5)	15.8 (6.1–34.9)	.01*	16.5 (5.8–33.4)	<.001*	.00*
**Parotid ipsi**	D_mean_ [Gy]	31.5 (13.3–48.4)	27.0 (12.4–46.2)	.00*	26.6 (11.1–45.5)	.00*	.00*
**SMG contra**	D_mean_ [Gy]	28.6 (16.4–61.3)	27.8 (17.0–60.3)	.59	27.0 (17.7–60.8)	.59	.05
**SMG ipsi**	D_mean_ [Gy]	42.5 (20.0–64.6)	36.6 (19.0–63.7)	.14	35.0 (18.4–63.7)	.02*	.00*
**Oral cavity**	D_mean_ [Gy]	11.2 (3.4–24.7)	11.4 (4.6–22.8)	.77	12.7 (4.9–22.3)	.26	.02*
**PCM superior**	D_mean_ [Gy]	55.9 (44.9–68.6)	54.8 (44.1–67.0)	.01*	54.6 (44.1–66.9)	.01*	.57
**PCM medius**	D_mean_ [Gy]	32.1 (20.4–44.0)	33.2 (23.0–44.2)	.00*	32.8 (23.3–43.8)	.03*	.06
**PCM inferior**	D_mean_ [Gy]	9.1 (2.3–22.4)	10.6 (4.1–23.6)	.13	9.7 (3.2–24.9)	.48	.1
**Cricopharyngeal inlet**	D_mean_ [Gy]	7.6 (0–18.1)	7.6 (0–15.6)	.03*	6.1 (0–14.1)	.00*	.05*
**Larynx**	D_mean_ [Gy]	10.0 (2.2–25.4)	9.0 (3.6–27.5)	.74	9.5 (2.6–27.6)	.82	.23
**Mandible**	D_2%_ [Gy]	60.0 (32.3–69.9)	57.4 (31.8–67.4)	.00*	56.0 (35.7–67.1)	.01*	.96
	vw-max D_2%_ [Gy]	66.3 (40.4–71.8)	63.8 (42.8–69.0)	.00*	63.4 (44.8–69.1)	.01*	.52
**Body-CTVs**	V_2Gy_ [cm^3^]	3915 (2960–7523)	3597 (2790–4965)	<.001*	3703 (2956–5172)	.08	<.001*
	V_5Gy_ [cm^3^]	3287 (2476–6075)	2966 (2275–4255)	<.001*	3094 (2350–4359)	<.001*	<.001*
	D_mean_ [Gy]	6.1 (2.7–9.3)	5.5 (2.1–8.6)	.00*	5.6 (2.2–8.8)	.03*	<.001*
**NTCP Xerostomia**	Grade ≥ 2 [%]	35.5 (23.3–53.7)	34.2 (22.6–52.2)	.00*	32.4 (22.1–52.0)	<.001*	<.001*
	Grade ≥ 3 [%]	9.3 (5.8–16.4)	8.9 (5.6–15.7)	<.001*	8.4 (5.5–15.6)	<.001*	<.001*
**NTCP Dysphagia**	Grade ≥ 2 [%]	7.2 (4.0–11.7)	7.2 (4.3–11.2)	.69	7.4 (4.1–11.1)	.67	.91
	Grade ≥ 3 [%]	0.7 (0.4–2.0)	0.8 (0.4–1.9)	.39	0.8 (0.4–1.9)	.42	.66

+ Metrics are provided for nominal plans, unless stated otherwise by ‘vw-min’ or ‘vw-max’. SMG=Submandibular gland, PCM=Pharyngeal constrictor muscle.

## 3 Results

**Fig. 1:**
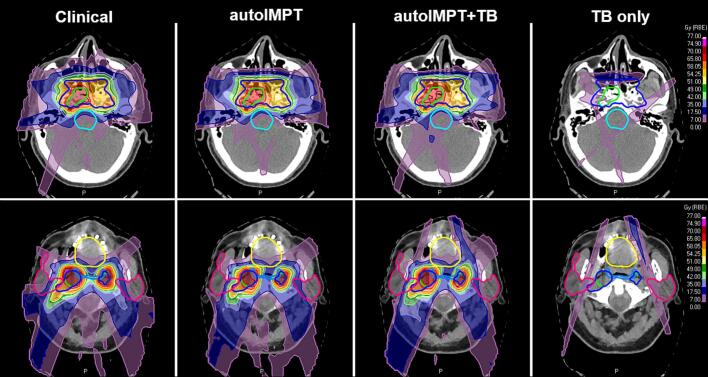
Transversal CT slices with typical dose distributions of the clinical, autoIMPT, autoIMPT+TB, and the TB-only contribution (11% of the total spots for this patient) to the autoIMPT+TB plan, for one patient (patient 11). Delineations: green=CTV7000, dark blue=CTV5425, light blue=brainstem, yellow=oral cavity, pink=parotids. In the automated plans, the brainstem and parotids receive less dose. (For interpretation of the references to colour in this figure legend, the reader is referred to the web version of this article.)

Fig. 1 shows a typical nominal dose distributions for one patient. The autoIMPT plan was similar to the clinical plan, with a steeper dose fall-off from the CTV. The autoIMPT+TB plan demonstrated enhanced lateral dose fall-off, resulting in lower brainstem dose, as shown in the dose difference maps (Supplementary Material, Fig. S1). Across all patients, the average contribution of the TBs to the IMPT+TB plans (number of TB spots/total number of IMPT+TB spots) was 14% (range 10–17%).

**Fig. 2:**
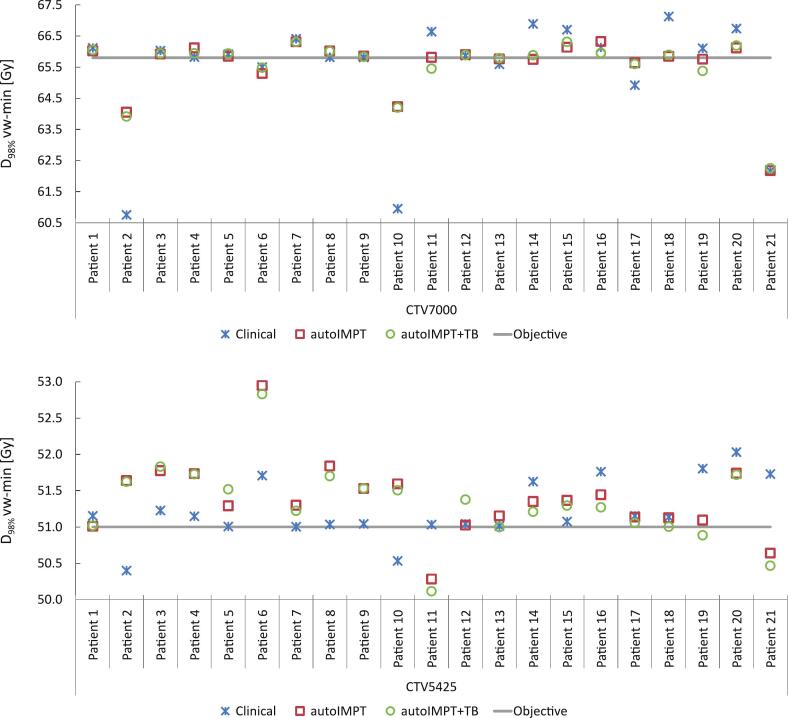
For all test patients, D_98%_ vw-min for CTV7000 (above) and CTV5425 (below) for the clinical, autoIMPT and autoIMPT+TB plans.

For both CTVs, no statistically significant differences in the median vw-min D98% were found between the automated and clinical plans (Table 2). For the vw-min dose, the D98%≥94% goal was not achieved for 6 patients in the clinical plans for CTV7000, whereas in the autoIMPT plan the coverage was improved for 4 of these patients, resulting in 16/21 patients with sufficient D98% vw-min for CTV7000, as shown in Fig. 2. For CTV5425, the D98%≥94% the vw-min dose was fulfilled in 19/21 patients in the autoIMPT plans, similar to the clinical plans, and for 18/21 patients in the autoIMPT+TB plans.

**Fig. 3:**
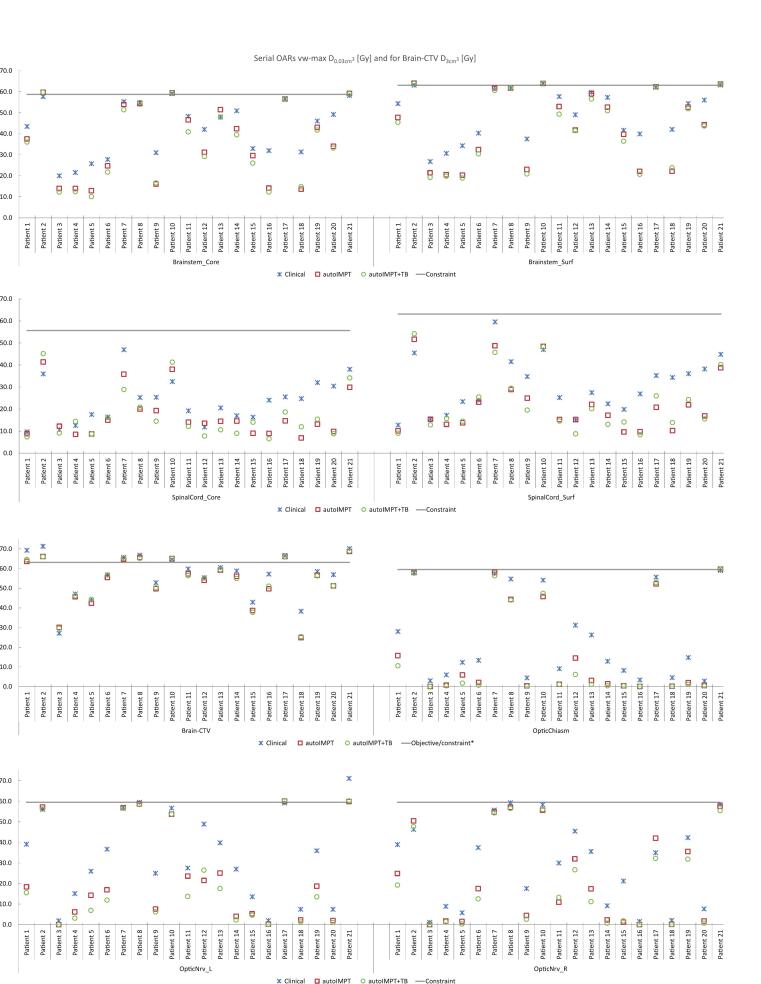
For all test patients, D_0.03__cm^3^_ in the serial OARs and D_3__cm^3^_ in the Brain-CTV in the vw-max dose for the clinical, autoIMPT and autoIMPT+TB plans. The grey line represents the constraint (*objective for Brain-CTV).

In all serial OARs, population median dose reductions in the automated plans were statistically significant (p < 0.05) as compared to clinical plans, for both the nominal and vw-max dose distributions (Table 2). Additional dose reductions in the autoIMPT+TB plans were observed as compared to the autoIMPT plans, statistically significant for the brainstem and optic system (last column Table 2) and dose reductions for individual patients results are shown in Fig. 3. Similar reductions in nominal serial OAR doses were observed (Supplementary Material, Fig. S2).

For all parallel OARs except the PCM medius, mean dose reductions were statistically significant (Table 2) and Supplementary Material Fig. S3 show Dmean reductions in individual patients. This is also reflected in the NTCP calculation for dysphagia which remained equal to that of the clinical plans, whereas for xerostomia statistically significant NTCP reduction was obtained in the autoIMPT (−1.3%. grade ≥ 2, and −0.4%. for grade ≥ 3) and autoIMPT+TB plans (−3.1%. grade ≥ 2, and −0.9%. for grade ≥ 3). The maximum dose per beam contribution was similar to clinical plans (Supplementary Material, Table A4).

## 4 Discussion

This study is to our knowledge the first that validates fully automated IMPT plan generation and automated IMPT plans in combination with transmission beams for NPC patients. Overall, the automated plans showed an advantage over the clinical IMPT plans in terms of dose/volume-based parameters, without hands-on planning time. It was hypothesized that combining IMPT and TB optimization would maximize the benefits of IMPT's steep distal dose fall-off and TB's small lateral penumbra. Indeed, we observed that in the autoIMPT+TB plans sparing in most serial OARs was more pronounced as compared to the autoIMPT plans.

In cases where clinical IMPT plans required target coverage concessions, automated plans improved coverage in most patients, although the inclusion of TBs did not yield further enhancement and, in some cases, slightly reduced coverage. This may be caused by the steeper lateral dose fall off of TBs, which could have contributed to less robustness in that direction as compared to IMPT. The slight increase in the oral cavity Dmean in the autoIMPT+TB plans can be explained by the shoot through dose originating from the TBs.

In the patients with a clinical target coverage underdosage, serial OAR constraints were limiting factors (Fig. 3). Automated plans achieved improved target coverage while maintaining these constraints, enabled by Erasmus-iCycle’s multi-criterial optimization, where prioritized objectives are sequentially optimized without violating constraints. The prioritization in our developed wish-list is in line with the international guidelines as described in Lee et al. [29], and as applied in our clinical protocol. For patient 21, the vw-min D98% for CTV5425 was lower than in the clinical plan, likely because the automated plan adhered to the left optic nerve constraint, whereas this was exceeded in the clinical plan. Unlike our method, the clinical planning system lacks hard constraints, and manual iterative adjustments on objective weights are required, which may not result in the optimal plan, is time-consuming, and depends on the planner’s expertise. Once clinical constraints are met, the planners will stop optimizing the plan further, whereas Erasmus-iCycle continues optimizing, further reducing OAR doses where possible. This resulted in the obtained additional sparing in the serial OARs in the automated plans, mostly at levels already far below the clinical constraints, but can be of clinical relevance in cases where future reirradiation is necessary. Furthermore, in case anatomical deformations occur during treatment [30], fully automated planning can be used to anticipate on these changes without hands-on planning time [31].

To be able to make a fair assessment of the added value of TB to IMPT, the same wish-list and beam configuration for the automated generation of IMPT+TB plans was used as for automatic generation of IMPT plans. To prevent undesirable beam broadening of the TBs, the range shifter was not used for the TB part in the autoIMPT+TB plans. For clinical practice, this might result in prolonged delivery times, however, this was outside the scope of our study. In addition, for TBs, an energy of 244 MeV was used, the highest energy currently commissioned in our institute. It would be interesting whether using higher TB energies will result in even smaller lateral penumbras to achieve high plan quality. Kong et al. reported the total plan generation time for IMPT+TB was on average 1.5 times longer than for IMPT [23].

Our results are in accordance to our previous study where fully automated IMPT plans were generated for oropharyngeal cancer cases, with similar target coverage and significantly improved OAR sparing compared to clinical IMPT planning[19]. Van Bruggen et al.[16] have used deep learning IMPT planning for oropharyngeal patients and Delaney et al.[13] used knowledge based planning for HNC cancer cases, non-NPC specific, both studies reported similar plan quality to clinical IMPT plans. Nevertheless, unlike to our approach, their methods depend heavily on the quality of the manually plans used as input.

However, Erasmus-iCycle for IMPT is currently not integrated yet in a clinical proton treatment planning system. Clinical implementation requires regulatory approval under the EU Medical Device Regulation (MDR), a process previously completed successfully for Erasmus-iCycle in photon therapy and brachytherapy [32].

There is an earlier study investigating joint TB-IMPT planning, but in the field of FLASH [33]. In three lung cases they found comparable CTV coverage, but higher OAR doses than in IMPT. Another study examined FLASH dose rates using TB alone in seven lung cancer patients, reporting inferior plan quality compared to IMPT [34]. Similarly, a study on hepatocellular carcinoma found TB-only plans to be suboptimal [7]. Unlike our IMPT+TB approach, these studies utilized TBs alone, limiting dose-shaping capabilities as in IMPT+TB and potentially compromising plan quality. Amstutz et al.[35] investigated combined proton–photon therapy for HNC, highlighting that the photon component was primarily employed to enhance dose gradients near critical structures, due to the sharper lateral penumbra. However, our IMPT+TB approach offers advantages by reduced planning complexity and logistical demands using a single-modality workflow.

Notably, in cases of target underdosage in autoIMPT, incorporating TBs with the current configuration and beam setup did not enhance coverage, and the additional serial OAR sparing may not be clinically relevant, limiting its benefits. Optimizing TB angles or increasing TB numbers, particularly parallel to target-OAR borders, may be necessary to maximize the benefit of the smaller lateral penumbras of TBs. Van Marlen et al.[10] already showed for a 10-beam TB only plan, similar OAR doses were achieved as in clinical 3-field IMPT plan for HNC. In addition, Fracchiolla et al.[36] demonstrated that clinically introduced proton arc therapy reduces doses to the brainstem and parallel OARs in HNC compared to 6-beam IMPT.

Currently, proton arc therapy is not supported in our treatment planning system, which prevents a direct comparison with IMPT+TB in this study. Nevertheless, future studies should evaluate the additional potential benefits of incorporating TBs into proton arc therapy. Combining the spatial freedom of arc delivery with the sharper lateral penumbras of TBs may further improve dose conformity and OAR sparing, whether through TB-only arc or a hybrid approach combining proton arc and TBs.

A limitation of our study is that LET calculations were not performed. Currently, in the clinically HNC IMPT plans, LET is not calculated and LET calculation is not yet implemented in Erasmus-iCycle. However, this should be performed in the future. In proton therapy, there are concerns about the increased radiobiological effect, caused by increased LET toward the distal edge of the Bragg peak, reaching maximum at the falloff region [37]. Luhr et al.[38] recommends to perform LET calculations, and use them in combination with dose for assessing clinical relevance. Even so, we expect lower LET in the TB part for the autoIMPT+TB plans of our study, since the end of the Bragg peak, where LET increase concerns are, is located outside of the patients.

In conclusion, fully automated robust IMPT and IMPT+TB plans can be generated with similar or improved target coverage and OAR dose compared to clinical manually created plans, especially for those NPC patients where concession to target coverage was needed in the clinical plan. The addition of the transmission beams to IMPT showed similar target coverage and enhanced dose reductions in serial OARs further.

## CRediT authorship contribution statement

**Merle Huiskes:** Conceptualization, Methodology, Software, Formal analysis, Investigation, Resources, Data curation, Writing – original draft, Writing – review & editing. **Wens Kong:** Software, Writing – review & editing. **Sebastiaan Breedveld:** Software, Resources, Writing – review & editing. **Koen Crama:** Resources, Writing – review & editing. **Martin de Jong:** Resources, Writing – review & editing. **Steven Habraken:** Conceptualization, Writing – review & editing. **Ben Heijmen:** Conceptualization, Writing – review & editing. **Coen Rasch:** Conceptualization, Methodology, Writing – review & editing, Supervision. **Eleftheria Astreinidou:** Conceptualization, Methodology, Writing – review & editing, Supervision.

## Declaration of competing interest

The authors declare that they have no known competing financial interests or personal relationships that could have appeared to influence the work reported in this paper.

## References

[1] Holliday E.B., Garden A.S., Rosenthal D.I., Fuller C.D., Morrison W.H., Gunn G.B. (2015). Proton Therapy Reduces Treatment-Related Toxicities for patients with Nasopharyngeal Cancer: a Case-Match Control Study of Intensity-Modulated Proton Therapy and Intensity-Modulated Photon Therapy. Int J Part Ther.

[2] Beddok A., Feuvret L., Noel G., Bolle S., Deberne M., Mammar H. (2019). Efficacy and toxicity of proton with photon radiation for locally advanced nasopharyngeal carcinoma. Acta Oncol.

[3] Paganetti H., Bortfeld T. (2006). Proton Therapy. New Technol. Radiat Oncol.

[4] Safai S., Bortfeld T., Engelsman M. (2008). Comparison between the lateral penumbra of a collimated double-scattered beam and uncollimated scanning beam in proton radiotherapy. Phys Med Biol.

[5] Taheri-Kadkhoda Z., Björk-Eriksson T., Nill S., Wilkens J.J., Oelfke U., Johansson K.A. (2008). Intensity-modulated radiotherapy of nasopharyngeal carcinoma: a comparative treatment planning study of photons and protons. Radiat Oncol.

[6] Lewis G.D., Holliday E.B., Kocak-Uzel E., Hernandez M., Garden A.S., Rosenthal D.I. (2016). Intensity-modulated proton therapy for nasopharyngeal carcinoma: Decreased radiation dose to normal structures and encouraging clinical outcomes. Head Neck.

[7] Wei S., Lin H., Choi J.I., Press R.H., Lazarev S., Kabarriti R. (2022). FLASH Radiotherapy using Single-Energy Proton PBS Transmission Beams for Hypofractionation Liver Cancer: Dose and Dose Rate Quantification. Front. Oncol.

[8] van Marlen P, van de Water S, Dahele M, Slotman BJ, Verbakel WFAR. Single Ultra-High Dose Rate Proton Transmission Beam for Whole Breast FLASH-Irradiation: Quantification of FLASH-Dose and Relation with Beam Parameters. Cancers (Basel) 2023;15. https://doi.org/10.3390/CANCERS15092579. 10.3390/cancers15092579 PMC10177419 37174045

[9] Mascia A.E., Daugherty E.C., Zhang Y., Lee E., Xiao Z., Sertorio M. (2023). Proton FLASH Radiotherapy for the Treatment of Symptomatic Bone Metastases: the FAST-01 Nonrandomized Trial. JAMA Oncol.

[10] van Marlen P, Dahele M, Folkerts M, Abel E, Slotman BJ, Verbakel W. Ultra-high dose rate transmission beam proton therapy for conventionally fractionated head and neck cancer: Treatment planning and dose rate distributions. Cancers (Basel) 2021;13. https://doi.org/10.3390/CANCERS13081859/S1. 10.3390/cancers13081859 PMC8070061 33924627

[11] Berry S.L., Boczkowski A., Ma R., Mechalakos J., Hunt M. (2016). Interobserver variability in radiation therapy plan output: results of a single-institution study. Pract Radiat Oncol.

[12] Nelms B.E., Robinson G., Markham J., Velasco K., Boyd S., Narayan S. (2012). Variation in external beam treatment plan quality: an inter-institutional study of planners and planning systems. Pract Radiat Oncol.

[13] Delaney A.R., Verbakel W.F., Lindberg J., Koponen T.K., Slotman B.J., Dahele M. (2018). Evaluation of an Automated Proton Planning solution. Cureus.

[14] Taasti V.T., Hong L., Deasy J.O., Zarepisheh M. (2020). Automated proton treatment planning with robust optimization using constrained hierarchical optimization. Med Phys.

[15] Hedrick S.G., Petro S., Ward A., Morris B. (2022). Validation of automated complex head and neck treatment planning with pencil beam scanning proton therapy. J Appl Clin Med Phys.

[16] van Bruggen I.G., Huiskes M., de Vette S.P.M., Holmström M., Langendijk J.A., Both S. (2023). Automated Robust Planning for IMPT in Oropharyngeal Cancer patients using Machine Learning. Int J Radiat Oncol Biol Phys.

[17] Kouwenberg J., Penninkhof J., Habraken S., Zindler J., Hoogeman M., Heijmen B. (2021). Model based patient pre-selection for intensity-modulated proton therapy (IMPT) using automated treatment planning and machine learning. Radiother Oncol.

[18] Kong W., Oud M., Habraken S.J.M., Huiskes M., Astreinidou E., Rasch C.R.N. (2024). SISS-MCO: large scale sparsity-induced spot selection for fast and fully-automated robust multi-criteria optimisation of proton plans. Phys Med Biol.

[19] Huiskes M., Kong W., Oud M., Crama K., Rasch C., Breedveld S. (2024). Validation of fully Automated Robust Multicriterial Treatment Planning for Head and Neck Cancer IMPT. Int J Radiat Oncol Biol Phys.

[20] Breedveld S., Storchi P.R.M., Heijmen B.J.M. (2009). The equivalence of multi-criteria methods for radiotherapy plan optimization. Phys Med Biol.

[21] Breedveld S., Storchi P.R.M., Voet P.W.J., Heijmen B.J.M. (2012). ICycle: Integrated, multicriterial beam angle, and profile optimization for generation of coplanar and noncoplanar IMRT plans. Med Phys.

[22] Fredriksson A., Forsgren A., Hårdemark B. (2011). Minimax optimization for handling range and setup uncertainties in proton therapy. Med Phys.

[23] Kong W., Huiskes M., Habraken S.J.M., Astreinidou E., Rasch C.R.N., Heijmen B.J.M. (2024). Reducing the lateral dose penumbra in IMPT by incorporating transmission pencil beams. Radiother Oncol.

[24] Heijmen B., Voet P., Fransen D., Penninkhof J., Milder M., Akhiat H. (2018). Fully automated, multi-criterial planning for Volumetric Modulated Arc Therapy – an international multi-center validation for prostate cancer. Radiother Oncol.

[25] Kooy H.M., Clasie B.M., Lu H.M., Madden T.M., Bentefour H., Depauw N. (2010). A Case Study in Proton Pencil-Beam Scanning delivery. Int J Radiat Oncol Biol Phys.

[26] Riet AV t., Mak ACA, Moerland MA, Elders LH, Van Der Zee W. A conformation number to quantify the degree of conformality in brachytherapy and external beam irradiation: Application to the prostate. Int J Radiat Oncol Biol Phys 1997;37:731–6. https://doi.org/10.1016/S0360-3016(96)00601-3. 10.1016/s0360-3016(96)00601-3 9112473

[27] Korevaar E.W., Habraken S.J.M., Scandurra D., Kierkels R.G.J., Unipan M., Eenink M.G.C. (2019). Practical robustness evaluation in radiotherapy - a photon and proton-proof alternative to PTV-based plan evaluation. Radiother Oncol.

[28] Langendijk JA, Hoogeman MS, Monshouwer R, Verheij M. Landelijk Indicatie Protocol Protonentherapie (versie 2.2) (LIPPv2.2) HOOFD-HALSTUMOREN Landelijk Platform Protonentherapie (LPPT) Landelijk Platform Radiotherapie Hoofd-halstumoren (LPRHHT) Verstuurd naar leden LPPT en LPRHHT Goedgekeurd door ZiN 2019.

[29] Lee A.W., Ng W.T., Pan J.J., Chiang C.L., Poh S.S., Choi H.C. (2019). International Guideline on Dose Prioritization and Acceptance Criteria in Radiation Therapy Planning for Nasopharyngeal Carcinoma. Int J Radiat Oncol Biol Phys.

[30] Huiskes M., Astreinidou E., Kong W., Breedveld S., Heijmen B., Rasch C. (2023). Dosimetric impact of adaptive proton therapy in head and neck cancer – a review. Clin Transl Radiat Oncol.

[31] Oud M., Breedveld S., Giżyńska M., Chen Y.H., Habraken S., Perkó Z. (2024). Dosimetric advantages of adaptive IMPT vs. Enhanced workload and treatment time – a need for automation. Radiother Oncol.

[32] Kolkman-Deurloo I.-K., Rossi L., Zolnay A., Christianen M., Westerveld H., Luthart L. (2024). In-House Developed Medical Device Software BiCycle for Automated BT Treatment Planning, Compliant with the European Medical Device Regulation (MDR). Brachytherapy.

[33] Lin Y., Lin B., Fu S., Folkerts M.M., Abel E., Bradley J. (2021). SDDRO-Joint: simultaneous dose and dose rate optimization with the joint use of transmission beams and Bragg peaks for FLASH proton therapy. Phys Med Biol.

[34] van Marlen P., Verbakel W.F.A.R., Slotman B.J., Dahele M. (2022). Single-fraction 34 Gy Lung Stereotactic Body Radiation Therapy using Proton Transmission Beams: FLASH-dose Calculations and the Influence of Different Dose-rate Methods and Dose/Dose-rate Thresholds. Adv Radiat Oncol.

[35] Amstutz F., Krcek R., Bachtiary B., Weber D.C., Lomax A.J., Unkelbach J. (2024). Treatment planning comparison for head and neck cancer between photon, proton, and combined proton–photon therapy – from a fixed beam line to an arc. Radiother Oncol.

[36] Fracchiolla F., Engwall E., Mikhalev V., Cianchetti M., Giacomelli I., Siniscalchi B. (2025). Static proton arc therapy: Comprehensive plan quality evaluation and first clinical treatments in patients with complex head and neck targets. Med Phys.

[37] Chaudhary P., Marshall T.I., Perozziello F.M., Manti L., Currell F.J., Hanton F. (2014). Relative biological effectiveness variation along monoenergetic and modulated Bragg peaks of a 62-MeV therapeutic proton beam: a preclinical assessment. Int J Radiat Oncol Biol Phys.

[38] Lühr A., Wagenaar D., Eekers D.B.P., Glimelius L., Habraken S.J.M., Harrabi S. (2025). Recommendations for reporting and evaluating proton therapy beyond dose and constant relative biological effectiveness. Phys Imaging Radiat Oncol.

