# Supplementary material for: Validation of fully automated intensity-modulated proton therapy with and without transmission beams for nasopharyngeal cancer
Source: Phys Imaging Radiat Oncol. 2025 Aug 30;35:100831. doi: 10.1016/j.phro.2025.100831 (PMC12451356; doi:10.1016/j.phro.2025.100831)
Supplement: Supplementary Data 1 [file mmc1.pdf]

Supplementary Material

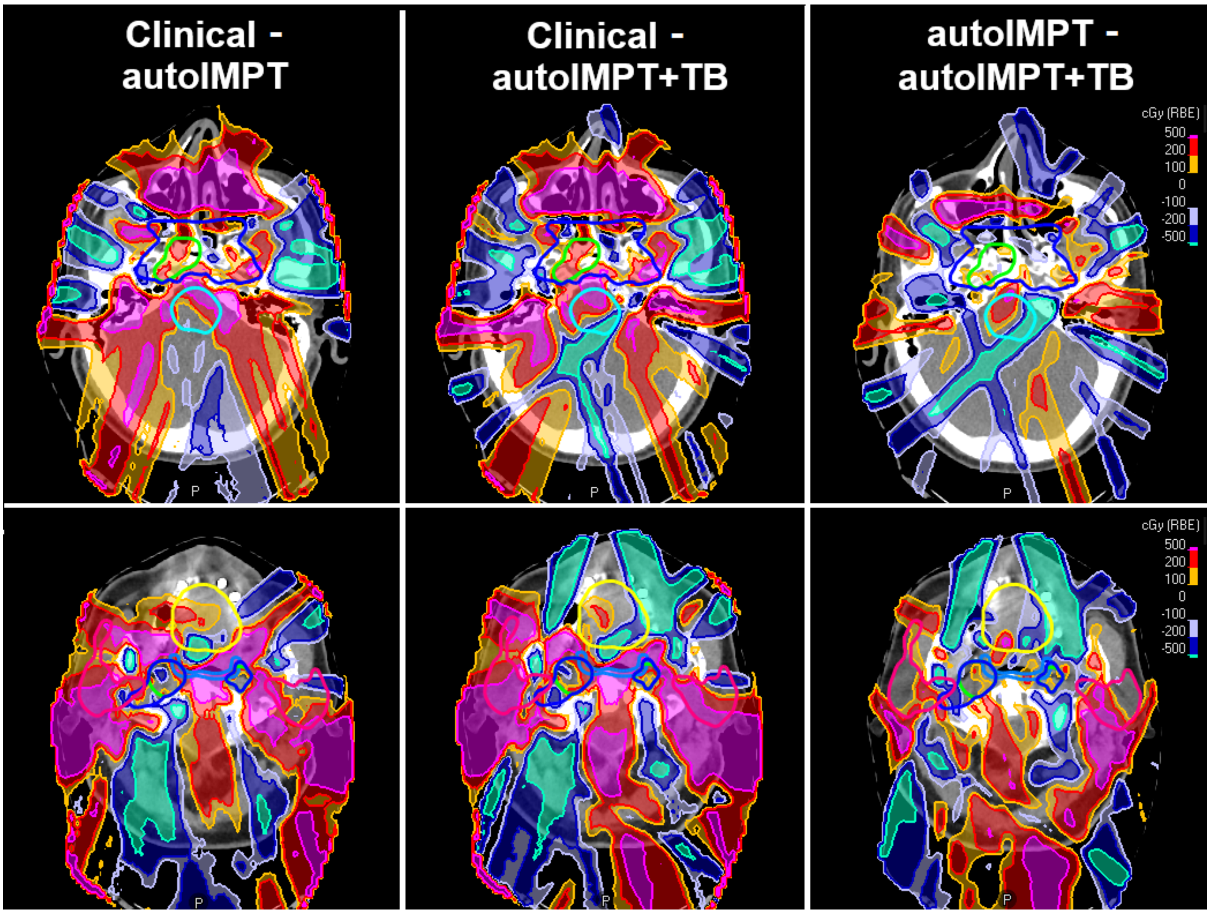

Figure S1 *Transversal CT slices showing dose differences between the clinical, autoIMPT and autoIMPT+TB plans from Figure 1. Delineations: green=CTV7000, dark blue=CTV5425, light blue=brainstem, yellow=oral cavity, pink=parotids. In the automated plans, less dose is received by the brainstem and parotids.*

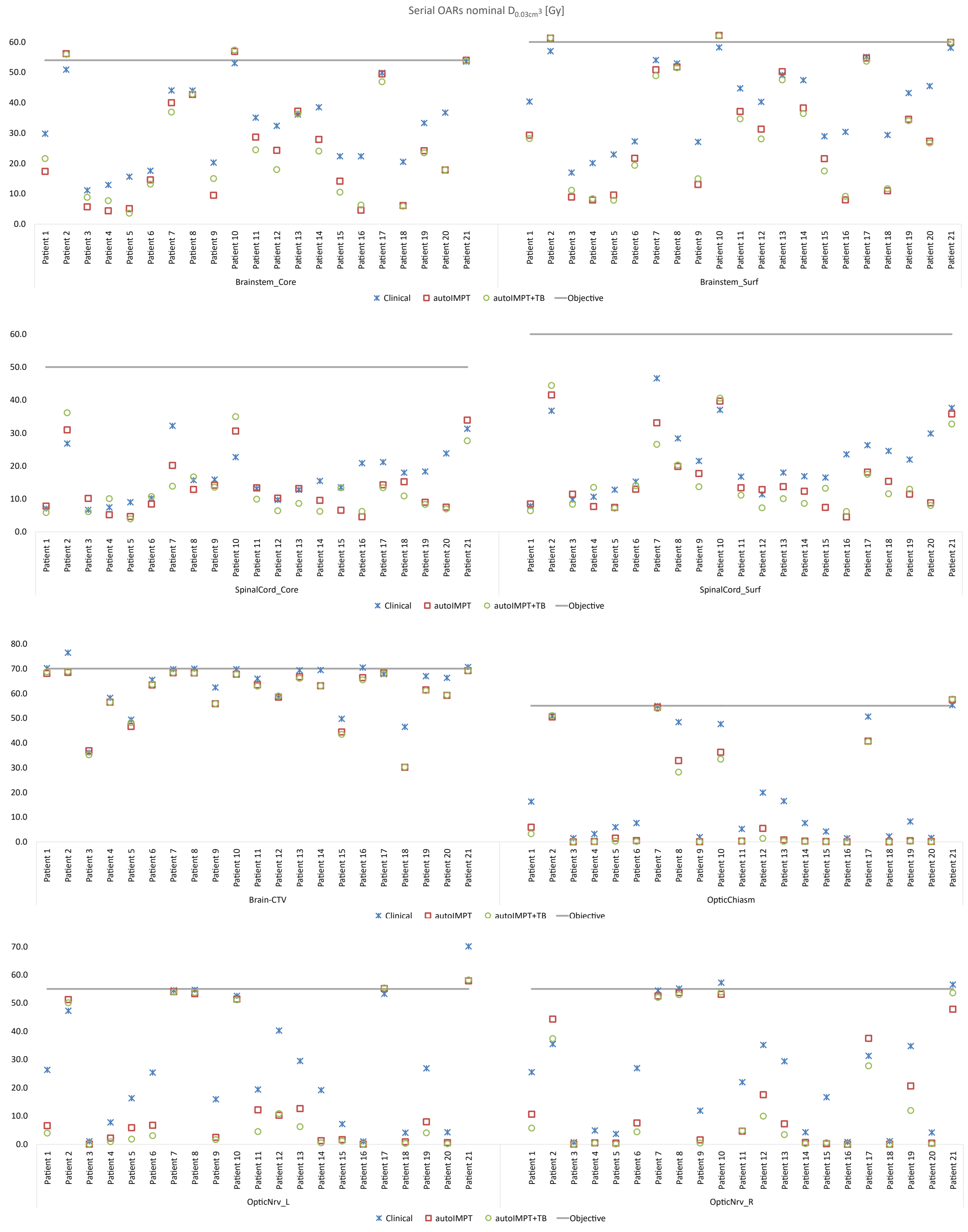

Figure S2 *For all test patients, $D_{0.03cm^3}$ in the serial OARs in the nominal dose for the clinical, autoIMPT and autoIMPT+TB plans. The grey line represents the objective.*

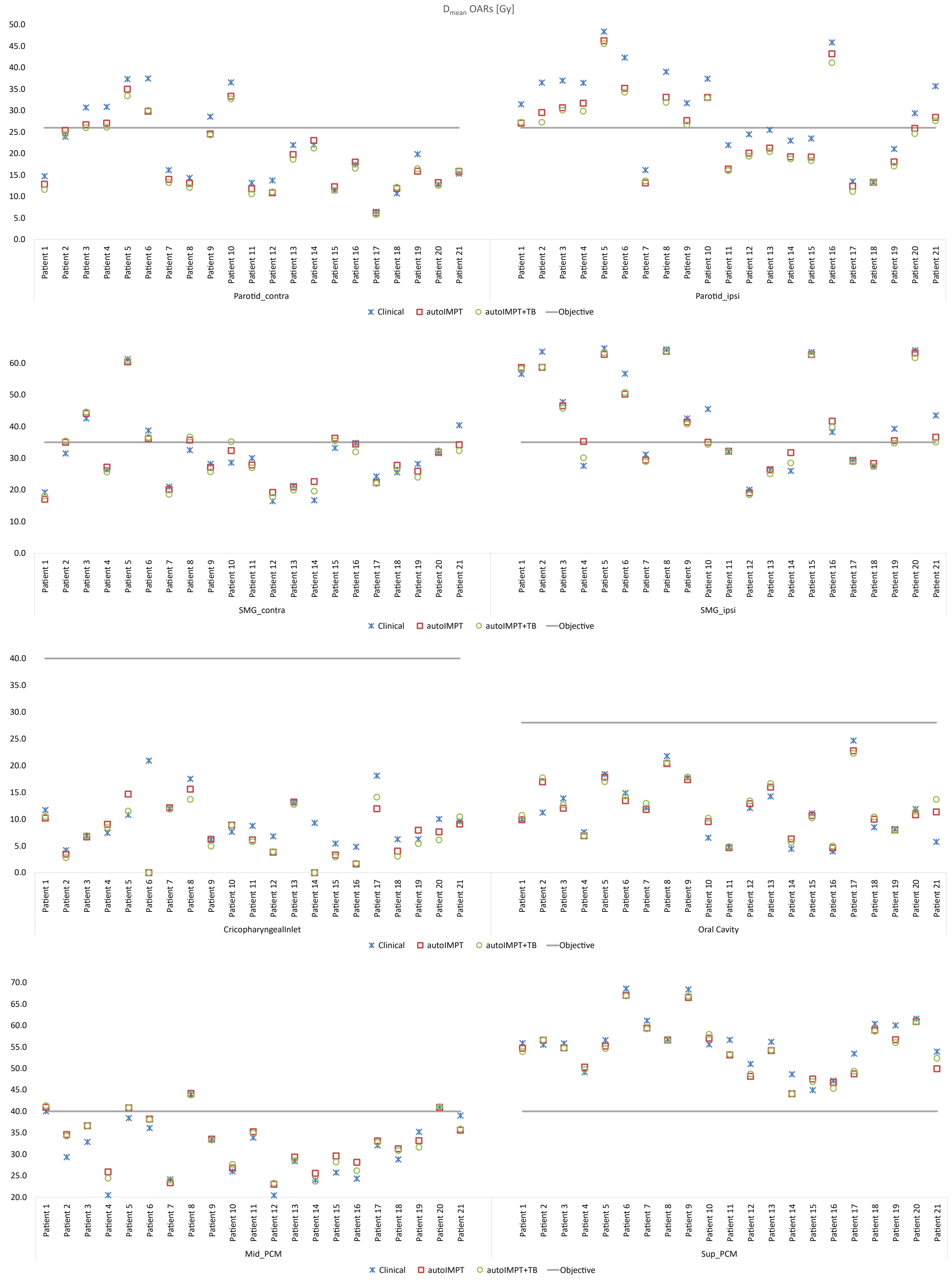
D$_{mean}$ OARs [Gy]
Parotid_contra
Parotid_ipsi
SMG_contra
SMG_ipsi
CricopharyngealInlet
Oral Cavity
Mid_PCM
Sup_PCM
Clinical
autoIMPT
autoIMPT+TB
Objective

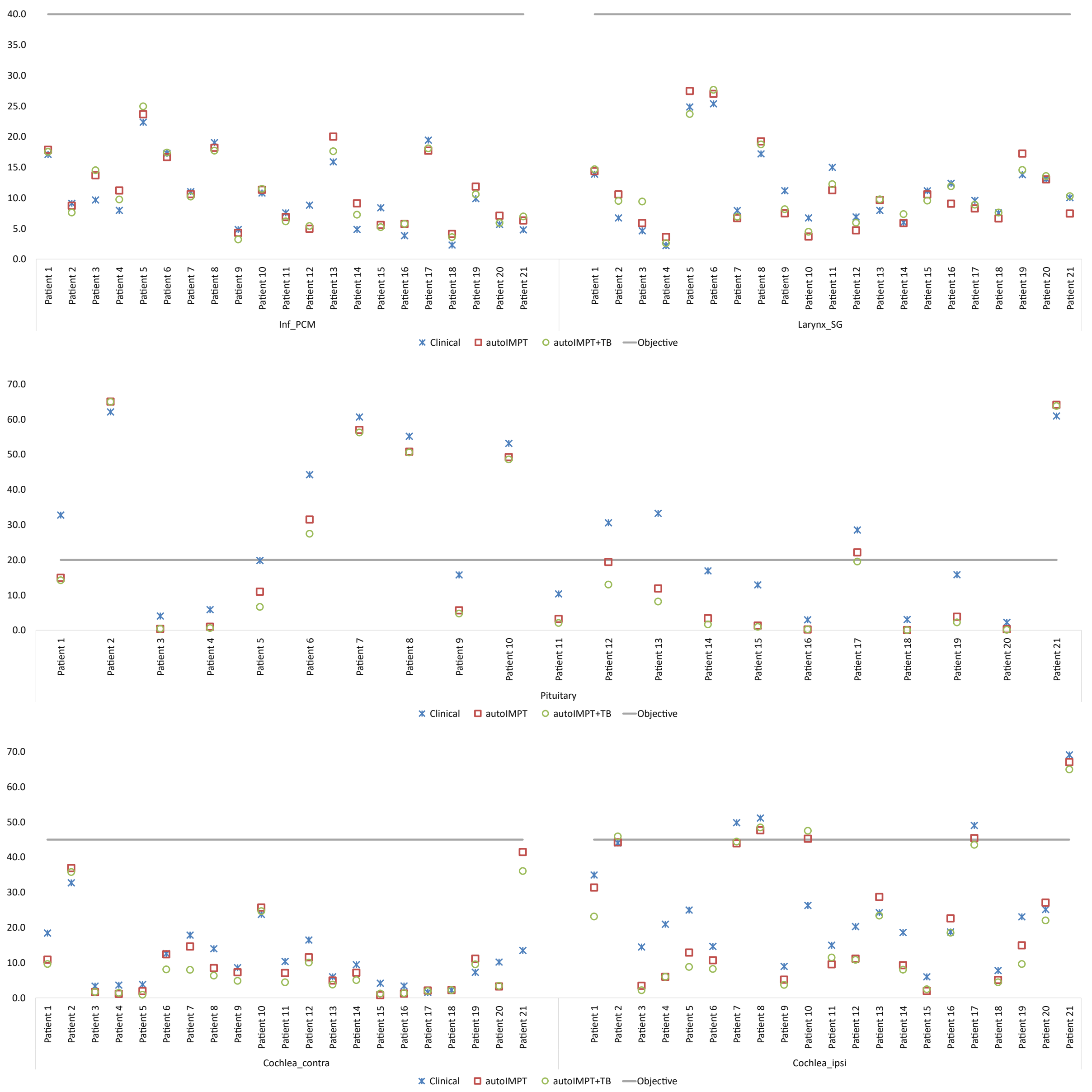

Figure S3 *For all test patients,* D_{mean} *to the parallel OARs in the nominal dose for the clinical, autoIMPT and autoIMPT+TB plans*

__Table S1__ Clinical treatment planning goals

Target/OAR		Dose volume goal	D_{mean}
**CTV7000**		$D_{98\%}$≥95%<br>vw-min $D_{98\%}$≥94%<br>$D_{2\%}$≤107%	
**CTV5425**		$D_{98\%}$≥95%<br>vw-min $D_{98\%}$≥94%	
**Brainstem**	Core	$D_{0.03cc}$≤54.0 Gy<br>**vw-max $D_{0.03cm^3}$≤58.7 Gy**	
	Surface	$D_{0.03cm^3}$≤60.0 Gy<br>**vw-max $D_{0.03cm^3}$cc≤63.1 Gy**	
**Spinal cord**	Core	$D_{0.03cm^3}$≤50.0 Gy<br>**vw-max $D_{0.03cm^3}$≤55.7 Gy**	
	Surface	$D_{0.03cm^3}$≤60.0 Gy<br>**vw-max $D_{0.03cm^3}$≤63.1 Gy**	
**Optic chiasm and optic nerves**		$D_{0.03cm^3}$≤55.0 Gy<br>**vw-max $D_{0.03cm^3}$≤59.5 Gy**	
**Eyes**			≤20.0 Gy
**Lenses**		$D_{0.03cm^3}$≤10.0 Gy<br>**vw-max $D_{0.03cc}$≤19.3Gy**	
**Brain-CTV7000**		**$D_{0.03cm^3}$≤70.0 Gy**<br>vw-max D_{3cm^3}≤63.1 Gy	
**Pituitary**			≤20.0 Gy
**Cochleae**			≤45.0 Gy
**Parotids**			≤26.0 Gy
**Submandibular glands**			≤35.0 Gy
**Oral cavity**			≤28.0 Gy
**PCMs**			≤40.0 Gy
**Cricopharyngeal inlet**			≤40.0 Gy
**Larynx**			≤40.0 Gy
**Mandible**		$D_{2\%}$≤70.0 Gy	

PCMs: Pharyngeal constrictor muscles (delineated separately the superior, medius and inferior part)
Bold goals are constraints

***Table S2** The nasopharynx specific wish-list used in this study*

Constraints					
	Structure	Constraint function	Type	Constraint	Robust
	CTV7000	↓ Maximum Dose	Linear	76.3 Gy	Yes
	CTV5425	↓ Maximum dose	Linear	59.7 Gy	Yes
	0-10mm ring around CTV_combined	↓ Maximum dose	Linear	57.5 Gy	Yes
	10-15mm ring around CTV_combined	↓ Maximum dose	Linear	48.8 Gy	Yes
	Brainstem Core	↓ Maximum Dose	Linear	58.7 Gy	Yes
	Brainstem Surface	↓ Maximum Dose	Linear	63.0 Gy	Yes
	Spinal cord Core	↓ Maximum Dose	Linear	55.7 Gy	Yes
	Spinal cord Surface	↓ Maximum Dose	Linear	63.1 Gy	Yes
	Optic Chiasm	↓ Maximum Dose	Linear	59.5 Gy	Yes
	Optic nerves	↓ Maximum Dose	Linear	59.5 Gy	Yes
	Mandible	↓ Maximum Dose	Linear	70.0 Gy	Yes
	Brain-CTV_7000	↓ Maximum Dose	Linear	69.0 Gy	
	Opt_Body	↓ Maximum Dose	Linear per beam	49.5 Gy	
	MU per spot	↓ Maximum Dose	Linear	80	

Objectives					
Priority	Structure	Aim & objective function	Type	Goal value (Sufficient[#])	Robust
1	CTV7000	↑ Minimum Dose	QUOP	67.2 Gy, α -0.2	Yes
1	CTV5425	↑ Minimum Dose	QUOP	51.5 Gy, α -0.2	Yes
2	CTV7000	↑ Minimum Dose	Linear	67.2 Gy	Yes
2	CTV5425	↑ Minimum Dose	Linear	51.5 Gy	Yes
2	CTV5425	↓ Maximum Dose	Linear	69.0 Gy (51.5)	Yes
3	Oral cavity	↓ Maximum Dose	Mean	28.0 Gy (1.0 Gy)	
3	Parotids	↓ Maximum Dose	Mean	26.0 Gy (1.0 Gy)	
3	Submandibular glands	↓ Maximum Dose	Mean	35.0 Gy (1.0 Gy)	
4	PCMs	↓ Maximum Dose	Mean	40.0 Gy (1.0 Gy)	
5	Larynx	↓ Maximum Dose	Mean	40.0 Gy (1.0 Gy)	
5	Esophagus	↓ Maximum Dose	Mean	40.0 Gy (1.0 Gy)	
5	Cricopharyngeal Inlet	↓ Maximum Dose	Mean	40.0 Gy (1.0 Gy)	
6	Cochlea	↓ Maximum Dose	Mean	44.0 Gy (1.0 Gy)	
7	Brain	↓ Maximum Dose	Mean	63.0 Gy (60.0 Gy)	
7	Pituitary	↓ Maximum Dose	Mean	20.0 Gy (1.0 Gy)	
7	Brainstem Core	↓ Maximum Dose	Linear	54.0 Gy	
7	Brainstem Surface	↓ Maximum Dose	Linear	60.0 Gy	
7	Spinal cord Core	↓ Maximum Dose	Linear	50.0 Gy	
7	Spinal cord Surface	↓ Maximum Dose	Linear	60.0 Gy	
8	Optic Chiasm	↓ Maximum Dose	Linear	54.9 Gy	
8	Optic nerves	↓ Maximum Dose	Linear	54.9 Gy	
8	Eyes	↓ Maximum Dose	Linear	19.0 Gy	
8	Lenses	↓ Maximum Dose	Linear	9.0 Gy	
9	All constraint OARs	↓ Maximum Dose	Mean	100.0 Gy (1.0 Gy)	
10	0-10 mm ring around CTV_combined	↓ Maximum Dose	Mean	1.0 Gy	
10	10-15 mm ring around CTV_combined	↓ Maximum Dose	Mean	1.0 Gy	
10	15-25 mm ring around CTV_combined	↓ Maximum Dose	Mean	1.0 Gy	
11	Opt_Body	↓ Maximum Dose	Linear per beam	47.0 Gy	

[#] = If no value; sufficient value is the goal value, ↓ = minimization, ↑ = maximization, CTV_combined = CTV5425 combined with CTV7000+10mm, PCMs: Pharyngeal constrictor muscles (delineated separately the superior, medius and inferior)

Table S3 *Regression coefficients for primary tumors for calculations of the normal tissue complication probability (NTCP) according to the Dutch National Indication Protocol for Proton Therapy.*[1]

**Variables**	**Endpoint (6 Months after Radiotherapy)**			
	**Xerostomia Grade ≥2**	**Xerostomia Grade ≥3**	**Dysphagia Grade ≥2**	**Dysphagia Grade ≥3**
**Constant (B0)**	−2.2951	−3.7286	−4.0536	−7.6174
**Slopes**				
√Dmean Parotid ipsilateral + √Dmean Parotid contralateral	0.0996	0.0855		
Dmean submandibular bilateral	0.0182	0.0156		
Dmean Oral cavity			0.0300	0.0259
Dmean PCM superior			0.0236	0.0203
Dmean PCM medius			0.0095	0.0303
Dmean PCM inferior			0.0133	0.0341
**Baseline score**				
Baseline xerostomia: None (EORTC QLQ-H&N35—Q41: score 1)	0.0000	0.0000		
Baseline xerostomia: A little (EORTC QLQ-H&N35—Q41: score 2)	0.4950	0.4249		
Baseline xerostomia: Quite (EORTC QLQ-H&N35—Q41: score 3–4)	1.2070	1.0361		
Baseline grade 0–1 dysphagia (normal foods)			0.0000	0.0000
Baseline grade 2 dysphagia (soft foods)			0.9382	0.5738
Baseline grade 3–4 dysphagia (liquid foods or TFD)			1.2900	1.4718
**Tumor location**				
Oral Cavity			0.0000	0.0000
Pharynx			−0.6281	0.0387
Larynx			−0.7711	−0.5303

PCM: Pharyngeal constrictor muscle

The NTCP can be calculated with this formula:

$$NTCP = \left(1 + e^{-(Constant + \sum Slope \cdot D_{mean} + Baseline\ score + Tumor\ location)}\right)^{-1}$$

[1] Langendijk JA, Hoogeman MS, Monshouwer R VM. Landelijk Indicatie Protocol Protonentherapie (versie 2.2) (LIPPv2.2) HOOFD-HALSTUMOREN 2019. https://nvro.nl/publicaties/rapporten (accessed February 24, 2023).

Table S4 *Maximum beam contribution as $D_{0.03cm^3}$ to Body [median (min-max)]*

Beam	Clinical plan [Gy]	auto IMPT plan [Gy]	auto IMPT+TB plan [Gy]
**50°**	46.2 (29.3-49.8)	47.1 (43.1-47.3)	47.1 (46.0-47.2)
**100°**	37.3 (26.7-47.5)	33.4 (22.4-44.4)	33.0 (21.4-44.5)
**160°**	37.1 (30.2-44.3)	42.2 (31.4-47.1)	45.7 (37.3-47.1)
**200°**	38.8 (27.8-46.1)	46.8 (34.7-47.3)	47.0 (37.0-47.2)
**260°**	35.2(26.4-46.8)	31.5 (23.1-40.2)	34.3 (23.1-44.1)
**310°**	43.8(31.1-49.6)	47.1 (28.9-47.3)	47.0 (32.7-47.2)
